# The Foggy Lungs: A Case Report on Pulmonary Alveolar Proteinosis

**DOI:** 10.7759/cureus.38150

**Published:** 2023-04-26

**Authors:** Gokul Paidi, Jugroop S Brar, Andrea S Vizcaino Duran, Jose Benero-Fossatti, Anhad Brar, Farrah P Aziz Greye

**Affiliations:** 1 Pulmonary and Critical Care Medicine, Arizona Lung, Sleep and Valley Fever Institute, Surprise, USA; 2 Medical Surgery, Universidad Autonoma de Guadalajara, Guadalajara, MEX; 3 General Medicine, Universidad Autonoma de Guadalajara, Guadalajara, MEX

**Keywords:** machrophage, granulocyte stimulating factor, lavage, alveoli, pulmonary alveolar proteinosis

## Abstract

Pulmonary alveolar proteinosis (PAP) is a rare interstitial lung disease characterized by macrophage dysfunction leading to the accumulation of surfactant in the alveoli and bronchiolar spaces, leading to impaired gas exchange and severe hypoxemia. The underlying mechanisms of PAP are not fully understood, but it is believed to involve impaired clearance of surfactant and abnormal immune responses. Diagnosis of PAP typically involves imaging studies and bronchoscopy, and treatment options include whole-lung lavage, pharmacotherapy, and lung transplantation. We report PAP in a 56-year-old female who worked in a dental office and had no prior diagnosis of lung disease.

## Introduction

Pulmonary alveolar proteinosis (PAP) is a rare interstitial lung disease characterized by macrophage dysfunction leading to the accumulation of surfactant in the alveoli and bronchiolar spaces, resulting in impaired gas exchange and severe hypoxemia [1-3]. The lipo-proteinaceous material is composed mainly of surfactant phospholipid and apoproteins. PAP can be caused by a spectrum of disorders that negatively affect surfactant production and clearance by alveolar macrophages [2,3].

The clinical features of PAP include an insidious onset with an indolent course of symptoms, such as asymptomatic presentation, dyspnea on exertion, cough, sputum production, fatigue, weight loss, and low-grade fever. Physical examination may be normal in the initial stages, but crackles, clubbing, and cyanosis may be observed in some patients [4,5].

## Case presentation

**Figure 1:**
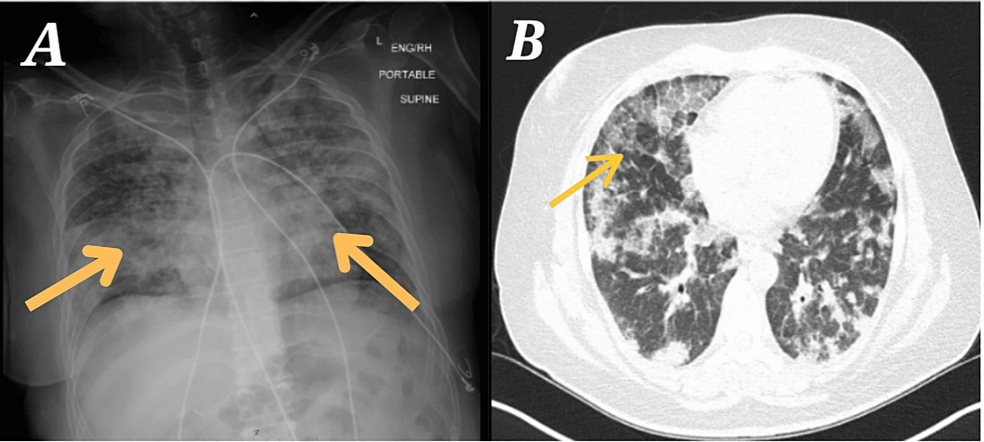
Chest X-ray and CT images of the patient's lungs A: Chest X-ray showing hazy opacities; B: CT scan showing crazy paving pattern

A 56-year-old Hispanic female with past medical history of type 2 diabetes mellitus and hypertension with no prior history of cigarette smoking or chronic lung disease presented with progressive productive cough for 10 months. She was COVID-19 vaccinated and has never had a prior COVID-19 infection. Patient denied known contacts with active Tuberculosis (TB) and worked in a dental office as intake staff, where she reported possible mold exposure. She had a long-standing productive cough with foul smelling thick yellow sputum, shortness of breath that worsend on exertion, throat pain and lightheadedness. Auscultation of the lung fields revealed prominent crackles throughout, with greater intensity in lung bases. Patient was initially treated with azithromycin and later with levofloxacin for suspected pneumonia. Chest X-ray showed extensive diffuse bilateral pulmonary infiltrates (Figure 1). She reported no improvement with antibiotics as well as minimal improvement in breathing with long acting beta agonists/long acting muscarinic and inhaled corticosteroids, clubbing of the fingernails was noted. Further lung evaluation was needed, so chest CT was ordered. CT demonstrated extensive bilateral pulmonary opacities with diffuse ground-glass opacities and alveolar infiltrates with a crazy paving pattern (Figure 1) in the upper lobes and right middle lobe [3-5]

The opacities in the superior segment of the right lower lobe appeared more confluent and consolidative while the opacities at the left lower lobe basilar segments were more nodular in appearance. Differential considerations were broad and included but did not limit infection, acute respiratory distress syndrome (ARDS), hypersensitivity pneumonitis, sarcoidosis, eosinophilic pneumonia, pulmonary edema, pulmonary alveolar proteinaceous cysts, or malignancy.

**Figure 2:**
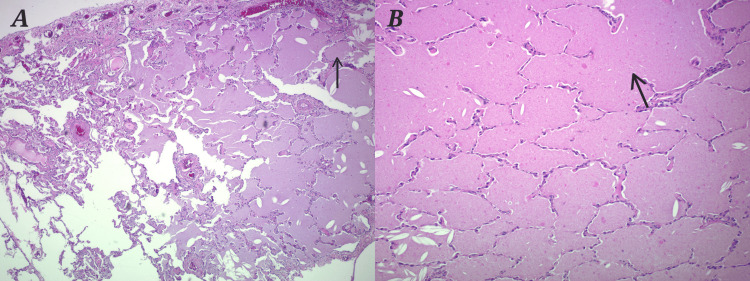
H&E stain showing alveoli filled with proteinaceous material A: Under low power showing proteinaceous material in alveoli; B: Under high power showing proteinaceous material in alveoli

The patient was given levofloxacin dexamethasone and put on oxygen via a nasal cannula. Although the patient reported mild improvement with antibiotics, steroids and continuous oxygen, she continued to have a foul smelling productive cough of cottage cheese consistency. Patient was then referred for bronchoscopy. At bronchoscopy, the mucosa and bronchial anatomy were normal, with no lesions or secretions. Bronchoalveolar lavage (BAL) was performed in the right middle lobe, and transbronchial brushing and biopsies were performed in the right lower lobe. The BAL fluid was clear, and samples were tested for cell count, bacterial, viral, fungal, acid-fast bacteria (AFB) analysis, and cytology. All cultures were negative. The pathology report showed amorphous eosinophilic debris in the alveolar spaces and a small focus on organizing pneumonitis with chronic inflammation and cholesterol cleft-containing giant cells. These findings were consistent with pulmonary alveolar proteinosis (PAP) (Figure 2).

Sargramostim, a granulocyte-macrophage colony-stimulating factor (GM-CSF), was prescribed. Most of the cases of alveolar proteinosis are caused by autoantibodies against GM-CSF. Secondary causes can be related to immunodeficiency. The patient did not have an immunodeficiency syndrome. We decided to treat her with subcutaneous sargramostim and prednisone. After two months, the patient had increased breathing difficulty whole lung lavage was performed after the procedure and condition of patient improved. After another two months, while still on sargramostim and prednisone, the patient was hospitalized for altered mental status and was found to have pneumonia, COVID-19, acute pancreatitis, diabetic ketoacidosis, and acute pancreatitis. She also had acute esophageal necrosis syndrome, deep vein thrombosis, and refractory shock. Unfortunately her condition did not improve despite vigorous treatment for two weeks, so she was moved to comfort care where she passed away.

## Discussion

**Figure 3:**
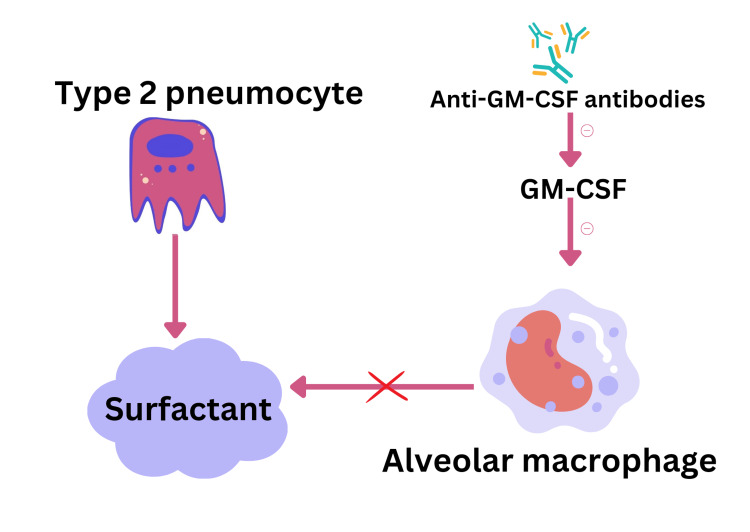
Pathogenesis of PAP Image credits: Gokul Paidi PAP: Pulmonary alveolar proteinosis

Pulmonary alveolar proteinosis (PAP) is a condition characterized by the accumulation of Periodic Acid-Schiff (PAS) positive lipo-proteinaceous material in distal airspaces, primarily composed of surfactant phospholipid and apoproteins. It is caused by disorders affecting surfactant production by alveolar cells and clearance by alveolar macrophages (Figure 3). There is little to no lung inflammation, and the underlying lung architecture remains preserved [6,7].

Patients with PAP often experience insidious onset and an indolent course of symptoms, including dyspnea on exertion, cough, and fatigue. Radiographic presentations of PAP feature bilateral symmetric alveolar opacities with a "bat-wing" appearance on chest X-ray and ground-glass opacities with a homogeneous distribution on CT scans [8,9].

Diagnosis of PAP involves an autoimmune panel and assessment of serum anti-GM-CSF antibodies. Treatment options include GM-CSF therapy administered via nebulizer or subcutaneous injection, plasmapheresis to remove GM-CSF autoantibodies, and whole lung lavage. Early diagnosis and appropriate treatment can improve outcomes and quality of life for patients with PAP [10,11].

## Conclusions

PAP is a rare interstitial lung disease characterized by the accumulation of surfactant in the alveoli and bronchiolar spaces, leading to impaired gas exchange and severe hypoxemia. This case highlights the importance of considering PAP in the differential diagnosis of patients presenting with chronic, unexplained respiratory symptoms and imaging findings consistent with the disease. Early diagnosis and appropriate treatment can lead to improved outcomes and quality of life for patients with PAP. Further research is needed to understand the pathophysiology better, improve diagnostics methods, and develop new treatment modalities to improve the prognosis in patients with PAP effectively.
